# Exposure to a Manuka Honey Wound Gel Is Associated With Changes in Bacterial Virulence and Antimicrobial Susceptibility

**DOI:** 10.3389/fmicb.2020.02036

**Published:** 2020-08-19

**Authors:** Jawahir A. Mokhtar, Andrew J. McBain, Ruth G. Ledder, Reem Binsuwaidan, Victoria Rimmer, Gavin J. Humphreys

**Affiliations:** ^1^Division of Pharmacy and Optometry, Faculty of Biology, Medicine and Health, School of Health Sciences, The University of Manchester, Manchester, United Kingdom; ^2^Faculty of Medicine, King Abdulaziz University, Jeddah, Saudi Arabia

**Keywords:** manuka honey, chronic wounds, MIC, biofilm, antimicrobials

## Abstract

The use of manuka honey for the topical treatment of wounds has increased worldwide owing to its broad spectrum of activity towards bacteria in both planktonic and biofilm growth modes. Despite this, the potential consequences of bacterial exposure to manuka honey, as may occur during the treatment of chronic wounds, are not fully understood. Here, we describe changes in antimicrobial susceptibility and virulence in a panel of bacteria, including wound isolates, following repeated exposure (ten passages) to sub-inhibitory concentrations of a manuka honey based wound gel. Changes in antibiotic sensitivity above 4-fold were predominantly related to increased vancomycin sensitivity in the staphylococci. Interestingly, *Staphylococcus epidermidis* displayed phenotypic resistance to erythromycin following passaging, with susceptibility profiles returning to baseline in the absence of further honey exposure. Changes in susceptibility to the tested wound gel were moderate (≤ 1-fold) when compared to the respective parent strain. In sessile communities, increased biofilm eradication concentrations over 4-fold occurred in a wound isolate of *Pseudomonas aeruginosa* (WIBG 2.2) as evidenced by a 7-fold reduction in gentamicin sensitivity following passaging. With regards to pathogenesis, 4/8 bacteria exhibited enhanced virulence following honey wound gel exposure. In the pseudomonads and *S. epidermidis*, this occurred in conjunction with increased haemolysis and biofilm formation, whilst *P. aeruginosa* also exhibited increased pyocyanin production. Where virulence attenuation was noted in a passaged wound isolate of *S. aureus* (WIBG 1.6), this was concomitant to delayed coagulation and reduced haemolytic potential. Overall, passaging in the presence of a manuka honey wound gel led to changes in antimicrobial sensitivity and virulence that varied between test bacteria.

## Introduction

Chronic wounds, such as diabetic foot ulcers, are associated with increased morbidity and mortality worldwide (Majtan et al., 2014). A wound is usually considered chronic if it has failed to heal within 8 weeks and leads to significant tissue loss as a result of disruption during the wound healing stages (McCarty et al., 2012). The impairment of wound healing is caused by a range of factors, with bacterial infection frequently cited as a major contributor and aggressive treatment is usually required (Edwards and Harding, 2004; Healy and Freedman, 2006; Lu et al., 2014). A rise in the number of antibiotic-resistant bacteria is a cause for concern in wound management and effective control must be accomplished (Bradshaw, 2011).

Honey has been reported to contain over 200 compounds, including sugars, vitamins, amino acids, minerals, enzymes, flavonoids, antioxidants and phenolic acids (Eteraf-Oskouei and Najafi, 2013; Schneider et al., 2013; Alvarez-Suarez et al., 2014; Stephens et al., 2015). Manuka honey (derived from the *Leptospermum scoparium* tree in New Zealand) is frequently applied in the treatment of bacterial infections (Qamar et al., 2017) and exhibits well documented antibacterial properties as a result of various phenolic compounds (Carter et al., 2016; Johnston et al., 2018) and methylglyoxal, the latter following inhibition of bacterial DNA and protein synthesis (Jervis-Bardy et al., 2011; Kilty et al., 2011; Hayes et al., 2018). At bactericidal concentrations, manuka honey has been reported to cause loss of membrane integrity in both Gram positive and negative bacteria, including *Pseudomonas aeruginosa* (Roberts et al., 2012). At subinhibitory concentrations, manuka honey has been shown to inhibit septa formation in the staphylococci (Henriques et al., 2010; Lu et al., 2013) and down regulation of flagella associated genes in pseudomonads (Roberts et al., 2014). This purported broad spectrum of activity offers some utility in the management of chronic wound infections. The microbiology of chronic wounds is complex and incompletely understood, although studies aiming to profile venous leg ulcers have identified both *Staphylococcus aureus* and *P. aeruginosa* in over 90 and 50% of samples, respectively (Davies et al., 2007; Han et al., 2011). Other taxa have also been reported, including *Enterococcus faecalis*, coagulase negative staphylococci, *Streptococcus* spp., members of the *Enterobacteriaceae* and anerobic rods (Han et al., 2011; Oates et al., 2012).

Concerns have been raised regarding co-selection for antibiotic resistance among bacteria exposed to non-antibiotic antimicrobial agents (McBain and Gilbert, 2001; Buffet-Bataillon et al., 2012; Wales and Davies, 2015). For example, laboratory exposure to some disinfectants has been shown to induce bacterial adaptations that may result in decreased susceptibility to one or more antibiotics (Chuanchuen et al., 2001; Forbes et al., 2014, 2015). Such changes may also occur in conjunction with other phenotypic adaptations that affect biofilm formation potential (Latimer et al., 2012; Henly et al., 2019), bacterial fitness (Forbes et al., 2015) and pathogenicity (Latimer et al., 2012; Bazaid et al., 2018; Henly et al., 2019). The effect of sub-lethal exposure to manuka honey has received relatively little research attention although stepwise training experiments using planktonic cultures of *Escherichia coli, P. aeruginosa, S. aureus* and *S. epidermidis* suggest only transient reductions in sensitivity to honey (Blair et al., 2009; Cooper et al., 2010). The anti-biofilm potential of manuka honey has been described (Maddocks et al., 2012; Lu et al., 2019; Roberts et al., 2019), although elevations in imipenem MIC of up to 4-fold and increased biofilm forming potential have been observed in cultures derived from honey exposed sessile communities (Camplin and Maddocks, 2014). Passaging experiments using other antimicrobials have demonstrated that adaptation can manifest in ways other than through changes in drug sensitivity (Latimer et al., 2012; Lu et al., 2014; Henly et al., 2019). Whilst the general understanding of such effects in response to manuka honey are less clear, a handful of gene expression studies support a view of virulence attenuation (Jenkins et al., 2014; Roberts et al., 2014). It must be noted, however, that manuka honey exhibits a complex mode of action that is capable of acting upon multiple cellular target sites with variable cellular responses to honey reported between different bacterial species (Jenkins and Cooper, 2012; Carter et al., 2016; Hayes et al., 2018; Johnston et al., 2018).

The present study aimed to investigate the consequences of bacterial passage in the presence of a manuka honey wound gel. Changes in biofilm formation, bacterial pathogenicity and exotoxin production were determined in conjunction with antimicrobial susceptibility profiling in planktonic and biofilm growth modes.

## Materials and Methods

### Bacteria

Wild-type clinical wound isolates were previously isolated from diabetic foot wounds as part of a previous study (Oates et al., 2014). These included: *Staphylococcus aureus* WIBG 1.2, *Staphylococcus aureus* WIBG 1.6, *Streptococcus pyogenes* WIBG 2.1, *Pseudomonas aeruginosa* WIBG 1.3, *Pseudomonas aeruginosa* WIBG 2.2 and *Escherichia coli* WIBG 2.4. Methicillin-resistant *Staphylococcus aureus* (MRSA) NCTC 11939 was obtained from the National Collection of Type Cultures (Public Health England, Salisbury, United Kingdom). *Staphylococcus epidermidis* ATCC 14990 was acquired from the American Type Culture Collection (LGC Standards, Teddington, United Kingdom).

### Chemicals and Media

All dehydrated bacteriological media were purchased from Oxoid (Basingstoke, United Kingdom) and autoclaved at 121°C (15 psi) for 15 minutes holding time prior to use. Medihoney^®^ antibacterial wound gel^TM^ (Derma Sciences, New Jersey, United States) was prepared as a 75% w/v stock solution in sterile distilled water before use. Medihoney^®^ wound gel^TM^ is formulated by the manufacturer and is stated to comprise Medihoney^®^ (80%) and waxes. Antibiotics were prepared as stock solutions (4000 mg/L) in distilled water for the purposes of sensitivity testing and sterilised through syringe filtration (0.22 μM; Milllipore, Watford, United Kingdom) before use. Clindamycin, erythromycin, fusidic acid, gentamicin, meropenem, tetracycline and vancomycin were obtained from Sigma-Aldrich (Dorset, United Kingdom). Ciprofloxacin was purchased from Alfa Aesar (Heysham, United Kingdom).

### Long-Term Exposure of Bacteria to Manuka Honey

Bacteria were repeatedly exposed to the manuka honey wound gel using an agar-based diffusion assay (Perez, 1990). In brief, 500 μL of 75% w/v wound gel solution was aseptically transferred into a 15 mm well, formed at the centre of a Mueller Hinton agar plate. Each parent strain of microorganism (P0) was distributed radially in triplicate around the central well and incubated at 37°C for 48 h. After incubation, the bacteria that exhibited growth at the innermost part of the radial streak were aseptically re-inoculated onto a fresh exposure plate. This procedure was repeated for a total of ten passages (P10). Since 500 μl of diluted wound gel was deposited in the well, the organisms were exposed to the wound gel on a concentration continuum from 75% (v/v) to effetively zero. The culture for the subsequent passage was sampled at the border between bacterial growth and inhibition and as such, the concentration was in all cases subinhibitory and therefore close to the MIC. To assess any permanent or transient changes in bacterial susceptibility, P10 strains were further subcultured a total of ten times in the absence of antimicrobial to create passage X10.

### Determination of Antimicrobial Sensitivitiy

The minimum inhibitory concentrations (MICs) and minimum bactericidal concentrations (MBCs) of bacteria were assessed using a broth microdilution method as described previously (Humphreys et al., 2011). Briefly, overnight bacterial cultures were adjusted to an OD_600_ of 0.8 and further diluted 1 in 100. Adjusted bacterial cultures were transferred to 96 well microtiter plates containing dilutions of wound gel solution that varied by 5% (w/v) intervals. Mueller Hinton broth was used as the growth medium for all sensitivity testing. Where MIC determinations were conducted for antibiotics, 100 μl of respective stock solution was transferred to 100 μl of double-strength Mueller Hinton broth to account for sock solutions being prepared in distilled water. Doubling dilutions were then prepared across the plate ordinate (2000–0 mg/L). The plates were incubated aerobically at 37°C for 18 h. The MIC was defined as the lowest concentration of antimicrobial to inhibit visible microbial growth after overnight incubation. To determine the MBC, aliquots of 5 μL were taken from the wells exhibiting no visible turbidity, spot plated onto the surface of a Mueller Hinton agar plate and further incubated overnight at 37°C. The lowest test concentration of antimicrobial that resulted in the absence of bacterial growth was reported as the MBC. Data are presented as means of biologically duplicated experiments, each comprising technical triplicates. Where doubling dilutions have been used, data are presented as geometric means.

### Minimum Biofilm Eradication Concentration (MBEC)

MBECs were determined using the MBEC assay^TM^ plate (Innovotech, Edmonton, Canada) (Ceri et al., 1999). Briefly, overnight bacterial cultures were adjusted to an OD_600_ of 0.8, then, further diluted 1 in 100 using sterile Mueller Hinton broth. 100 μL of adjusted bacterial inoculum was then transferred into each well of the MBEC assay^TM^ plate and incubated at 37°C for 48 h to support biofilm formation on the transposable pegs. Pegged lids were subsequently transferred to an antimicrobial challenge plate containing doubling dilutions of the applicable antibiotic and incubated for 24 h at 37°C. After incubation, the pegged lid was moved to a recovery plate that contained 200 μL of sterile Mueller Hinton broth and sonicated (50 kHz, 5 min) to detach cells from the transposable lid surface using a SC-52TH Sonicator (Sonicor, New York, United States). Recovery plates were incubated for 24 h at 37°C. The minimum biofilm eradication concentrations (MBECs) were defined as the lowest concentration of antibiotic required to eliminate the biofilm. Data are presented as means of biologically duplicated experiments, each comprising technical triplicates.

### Crystal Violet Biofilm Assay

The potential to form biofilms was compared in parent, P10 and X10 bacteria using a crystal violet assay. Overnight bacterial cultures were adjusted to an optical density of 0.8 and then diluted 1:100 in Mueller Hinton broth. Aliquots (150 μl) of diluted bacterial culture were transferred to the wells of a sterile 96-well microtiter plate (Corning Ltd., Weisbaden, Germany) and were incubated aerobically for 48 h at 37°C. After 48 h, the liquid in the wells was removed by inversion of the microtiter plate, and the wells were washed twice using 200 μl of sterile phosphate-buffered saline (PBS). The wells were stained with 250 μl of 1% (w/v) crystal violet solution for 1 min, rinsed twice with PBS and left to air dry at room temperature. To solubilise the attached crystal violet, 300 μl of absolute ethanol was added to each well (10 min) before measuring the absorbance (OD_600_) using a PowerWave^TM^ XS plate reader (BioTek, Swindon, United Kingdom). Data were presented as biofilm units calculated by dividing the absorbance of the crystal violet bound biofilm by a corresponding planktonic OD_600_ in order to adjust for planktonic mass. All data points were plotted and analysed using GraphPad Prism version 7.0 (GraphPad Software, California, United States) and are presented as means of biologically duplicated experiments, each comprising six technical repeats. Differences between parent and passaged bacteria (P0 vs. P10; P0 vs. X10) were determined using a Mann-Whitney test.

### *Galleria mellonella* Pathogenicity Assay

The methodology was performed as described previously (Latimer et al., 2012). Larvae of *Galleria mellonella* were purchased from Live Foods Direct (Sheffield, United Kingdom) and stored in the dark for a maximum of 7 d. For each treatment group, 10 larvae were randomly assigned and placed in Petri dishes. Overnight suspensions of P0, P10 and X10 bacteria were centrifuged (13,000 rpm, 10 min) and washed twice using sterile PBS. Then, bacterial suspensions were adjusted using a light spectrophotometer and corresponding CFUs determined through viable counting. Briefly, quantification was performed following 1 in 10 serial dilutions in Mueller Hinton broth. Diutions were plated in triplicate onto Mueller Hinton agar and incubated overnight (18 h; 37°C). The corresponding standard bacterial inoculae were as follows: *S. aureus* WIBG1.2 (OD_600_ = 0.1, 1.2 × 10^9^ CFU/ml); *S. aureus* WIBG1.6 (OD_600_ = 0.1, 1.3 × 10^9^ CFU/ml); MRSA (OD_600_ = 0.1, 1.6 × 10^9^ CFU/ml); *S. epidermidis* (OD_600_ = 0.1, 5.8 × 10^8^ CFU/ml); *S. pyogenes* (OD_600_ = 0.1, 1.4 × 10^8^ CFU/ml); *P. aeruginosa* WIBG1.3 (OD_600_ = 0.1 followed by 1:1000000 dilution, 250 CFU/ml), *P. aeruginosa* WIBG2.2 (OD_600_ = 0.1 followed by 1:1000000 dilution, 64 CFU/ml) and *E. coli* (OD_600_ = 0.1 followed by 1:500000 dilution, 1.9 × 10^4^ CFU/ml). These dilutions were determined following in-house testing in order to achieve observable kill rates across the 7 d test period. Each of the larvae were injected with 5μl of adjusted bacterial suspension into the hemocele via the last left proleg using a sterile Hamilton syringe (Sigma, Dorset, United Kingdom). Larvae were incubated in a petri dish at 37°C, and the number of surviving individuals recorded daily for up to 7 d. An untreated group (no injection) and a group injected with sterile PBS were used as controls. All experiments were performed as biological duplicates with each assay comprising 10 worms. Tests were terminated when two or more of the control larvae died. The data were presented as a Kaplan-Meier survival curves and intra-strain, pairwise comparisons of datasets (P0 vs. P10; P0 vs. X10) were conducted using the log-rank test in Graph Pad Prism 7 (GraphPad Software, California, United States).

### Planktonic Growth Rate

Overnight cultures of all bacterial isolates (P0, P10, and X10) were adjusted to an OD_600_ of 0.8, further diluted 1:100 in Mueller Hinton broth and deposited into 96 well plates. To determine the planktonic growth rate of bacteria, the culture plate was placed into a microplate reader (PowerWave^TM^ XS, BioTek, Swindon, United Kingdom) and the optical density was read every hour for 24 h using Gen5^TM^ 1.08 software (BioTek, Swindon, United Kingdom). Growth curve data from eight absorbance readings (biological duplicates each comprising 4 technical replicates) were fitted to a standard form of the logistic equation using the R software package Growthcurver (Sprouffske and Wagner, 2016) to determine metrics relating to intrinsic growth rates (*r;* h^–1^), carrying capacity (*K*) and maximum generation time (t_gen; h^–1^). Pairwise statistical comparisons of generated datasets were performed between parent and passaged mutants (P0 vs. P10; P0 vs. X10) at *P* ≤ 0.05 using a Wilcoxon signed-rank test. Comparisons were performed using SPSS version 22 (IBM analytics, New York, United States).

### Haemolysin Assay

Haemolytic activity was measured for all strains that showed a significant change in pathogenicity and exhibited haemolysis when grown in blood agar (Latimer et al., 2012). P0, P10 and X10 passaged bacteria were grown in Mueller Hinton Broth overnight at 37°C. The overnight cultures were diluted 1:100 and incubated at 37°C until an OD_600_ of 0.3 was achieved. Then, whole defibrinated horse blood (5% v/v; Oxoid Ltd., Basingstoke, United Kingdom) was added to the samples and also to sterile broth (negative control). All assay reactions were incubated in a shaking incubator (100 rpm, 37°C) for 3 h. 1 ml aliquots were then removed and centrifuged at 16,000 g for 4 min (1-14 Microfuge, Sigma-Aldrich, Dorset, United Kingdom). Optical density measurements of the supernatant were determined using a light spectrophotometer (540 nm). In order to control for variability in growth rates, haemolytic activity was adjusted according to viable counts. In order to do this, serial dilutions (1 in 10) were performed, plated onto mannitol salt agar and incubated overnight (18 h; 37°C). Percentage heamolysis was expressed as the change in *A*_540_ (Δ*A*_540_)/cfu. Statistical comparisons (P0 vs. P10; P0 vs. X10) were performed in GraphPad Prism version 7 (GraphPad Software, California, United States) using a student’s unpaired *T*-test with Welch’s correction. Data are presented as means from biologically replicated experiments (*n* = 4).

### Coagulase Assay

Overnight cultures of P0, P10, and X10 *S. aureus* (WIBG 1.2 and 1.6), MRSA and *S. epidermidis* were adjusted to an OD_600_ of 0.4. Aliquots (1 ml) were added to 3 ml of rabbit plasma with EDTA (Bactident coagulase, Merck, Darmstadt, Germany) and incubated at 37°C in a water bath. Tubes were examined for signs of coagulation over 3 h and scored on a five-point scale according to the manufacturer’s instructions. Assays were performed as biological triplicates. *S. epidermidis* ATCC 14990 was included a negative assay control.

### Pyocyanin Assay

Differences in pyocyanin production between parent, P10 and X10 bacteria were performed using a chloroform extraction approach in order to better understand differences in virulence potential before and after honey exposure. Pyocyanin was determined as described elsewhere (Essar et al., 1990). 10-ml of overnight bacterial culture was grown (37°C, 200 rpm for 24 h) in PB medium (20 g Bacto peptone, 1.4 g MgCL_2_, 10 g K_2_SO_4_) to maximize pyocyanin production. 6 ml of chloroform was added to 10 ml of cell-free supernatant and shaken vigorously until the pyocyanin was extracted into the chloroform layer. The chloroform layer was drawn off and vigorously mixed with 2 ml of 0.2 N HCL to give a pink to deep red solution. The absorbance of this extracted solution was measured at OD_520 nm._ The percentage of pyocyanin production was expressed relative to the parent strain as follows: *A*_520_ (Δ*A*_520_)/cfu. Viable counts from corresponding 24 h PB cultures were determined as described for the “*Galleria mellonella* assay.” Pyocyanin concentrations were were performed in biological triplicates and the data expressed as means using GraphPad Prism version 7 (GraphPad Software, California, United States). Comparisons between parent and passaged bacteria (P0 vs. P10; P0 vs. X10) were determined using an unpaired *T*-test with Welch’s correction.

## Results

### Manuka Honey Wound Gel Susceptibility in Passaged Bacteria

The data presented in Table 1 demonstrates the susceptibilities of all tested bacteria to manuka honey wound gel before and after passaging experiments. MICs varied between bacterial test species and strains, ranging from 7.5% (w/v) to 70% (w/v) following broth microdilution. Overall, 4/8 of bacteria tested exhibited a change in MIC when compared to the parent strain. These changes were marginal (≤ 1-fold vs. baseline) but sustained in the absence of additional gel passaging (X10), except for *S. aureus* WIBG 1.6 which returned to baseline levels.

**TABLE 1:** Bacterial sensitivities to a manuka honey wound gel before and after passaging.

Bacterium	MIC (%w/v)			MBC (%w/v)		
	(P0)	(P10)	(X10)	(P0)	(P10)	(X10)
*S. aureus* WIBG 1.2	15	15	15	30	30	30
*S. aureus* WIBG 1.6	15	7.5 (2.7)	15	30	15	30
MRSA	11.66 (2.5)	7.5 (2.7)	7.5 (2.7)	30	30	30
*S. epidermidis*	15	30	30	30	60	60
*S. pyogenes*	15	15	15	30	30	30
*P. aeruginosa* WIBG 1.3	60	70	70	>70	>70	>70
*P.aeruginosa* WIBG 2.2	30	30	30	>70	>70	>70
*E.coli*	30	30	30	>70	>70	>70

*Data are presented as means from biologically duplicated experiments each comprising technical triplicates. Standard deviations are given in parenthesis if the data varied between replciates.*

### Antibiotic Susceptibility in Passaged Bacteria

Table 2 illustrates the susceptibility of wound bacteria to tested antibiotics following passage. Changes in sensitivity to at least one antibiotic were observed in all bacteria. MIC fold changes of ≥ 4-fold to baseline were less frequent (5/8 bacteria) and typically associated with increased sensitivity of the staphylococci to vancomycin. Of note, *S. epidermidis* exhibited transient reductions in sensitivity to both erythromycin and tetracycline marked by a *c.* 7-fold and 31-fold increase in MIC, respectively. With regards to sessile communities, 7/8 bacteria exhibited changes in biofilm eradication concentration to at least one antibiotic following the investigation of honey passaged isolates in MBEC devices (Table 3). Overall, of the MBECs with observable endpoints in P10 bacteria, 14/19 exhibited no change or less than a 1-fold change to baseline data. Generally, changes in MBEC in excess of 4-fold were infrequent (2/8 bacteria). Both strains of *P. aeruginosa* reported increased MBECs towards gentamicin with strain WIBG 2.2 representative of a *c.* 7-fold reduction in sensitivity. In contrast, passage X10 of *S. aureus* WIBG 1.6 yielded a *c.* 6-fold increase in ampicillin sensitivity when cultured as a biofilm.

**TABLE 2:** Antibiotic susceptibilities of bacteria before and after passaging.

Bacterium	Antibiotics	MIC (mg/l)			MBC (mg/l)		
		P0	P10	X10	P0	P10	X10
*S. aureus* WIBG 1.2	Vancomycin	0.98	**0.12**	0.24	15.6	3.90	15.6
	Ciprofloxacin	0.24	0.24	0.24	0.98	0.49	0.98
	Erythromycin	0.49	0.98	0.98	15.6	31.25	15.60
	Fusidic acid	0.49	0.49	0.49	1.95	3.90	3.90
	Ampicillin	2000	1000	1000	ns	2000	2000
	Tetracycline	0.98	0.45	0.49	7.81	3.90	3.90
*S. aureus* WIBG 1.6	Vancomycin	0.98	**0.17 (0.06)**	0.49	0.98	1.95	1.95
	Ciprofloxacin	1.95	0.49	1.95	15.60	3.90	15.60
	Erythromycin	31.25	15.6	15.60	62.50	62.50	62.50
	Fusidic acid	31.25	15.6	31.25	125	62.50	62.50
	Ampicillin	2000	1000	1000	2000	2000	2000
	Tetracycline	0.24	0.24	0.35 (0.133)	1.95	1.95	1.95
MRSA	Ciprofloxacin	1.95	1.95	1.95	3.90	3.90	3.90
	Fusidic acid	0.12	0.12	0.12	0.98	1.95	1.95
	Ampicillin	2000	2000	2000	ns	2000	ns
	Vancomycin	0.98	**0.11**	0.24	3.90	1.95	1.95
S. *epidermidis*	Ciprofloxacin	0.98	0.49	0.49	1.95	0.98	0.98
	Fusidic acid	0.24	0.24	0.24	1.95	1.95	1.95
	Vancomycin	1.95	**0.29 (0.12)**	0.98	15.6	3.90	7.81
	Erythromycin	0.49	**15.6**	0.49	1.95	**15.60**	3.90
	Tetracycline	7.81	**62.50**	15.60	15.60	62.50	31.25
*S. pyogenes*	Ciprofloxacin	0.49	0.49	0.49	0.98	0.98	0.98
	Erythromycin	0.24	0.98	0.35 (0.14)	1.95	7.81	1.95
	Tetracycline	0.24	0.98	0.49	7.81	15.60	7.81
*P. aeruginosa* WIBG 1.3	Ciprofloxacin	0.29 (0.12)	0.24	0.24	0.98	0.49	0.98
	Gentamicin	0.98	3.90	1.95	1.95	7.81	1.95
	Meropenem	0.98	0.98	0.98	1.95	1.95	1.95
*P. aeruginosa* WIBG 2.2	Ciprofloxacin	0.03	**0.24**	0.03	0.24	0.98	0.24
	Gentamicin	0.98	0.49	0.98	3.90	3.90	3.90
	Meropenem	0.98	0.49	0.49	1.95	0.98	1.95
*E. coli* WIBG 2.4	Ciprofloxacin	0.02	0.02	0.02	0.12	0.24	0.24
	Gentamicin	0.98	3.90	1.95	3.90	15.60	3.90
	Meropenem	0.12	0.12	0.12	0.24	0.24	0.24

*Bold type indicates a ≥ 4-fold change when comparing baseline sensitivities (P0) to P10 and X10 values. Data are expressed as geometric means from biologically duplicated experiments, with each comprising technical triplicates. Standard deviations are given in the parentheses if the data varied between replicates. Non-susceptible (ns) denotes no sensitivity breakpoint determined as the value was in excess of the antimicrobial concentrations used in the broth dilution.*

**TABLE 3:** Biofilm eradication concentrations for parent and passaged bacteria.

Bacterium	Antibiotics	MBEC (mg/l)		
		P0	P10	X10
*S. aureus* WIBG 1.2	Ciprofloxacin	62.50	31.30	31.30
	Vancomycin	62.50	62.50	62.50
*S. aureus* WIBG 1.6	Ciprofloxacin	62.50	62.50	62.50
	Vancomycin	125	31.30	62.50
	Ampicillin	16000	4000	**2244 (816)**
MRSA	Ciprofloxacin	15.60	15.60	15.60
	Vancomycin	15.60	62.50	62.50
	Ampicillin	ns	ns	ns
*S. epidermidis*	Ciprofloxacin	7.81	11.03 (4.26)	7.81
	Vancomycin	62.50	31.25	62.50
*S. pyogenes*	Ciprofloxacin	3.90	3.90	3.90
*P. aeruginosa* WIBG 1.3	Ciprofloxacin	7.81	7.81	7.81
	Gentamicin	125	500	125
	Meropenem	31.30	15.60	15.60
*P. aeruginosa* WIBG 2.2	Ciprofloxacin	3.90	3.90	4.37 (1.59)
	Gentamicin	15.60	**125**	15.60
	Meropenem	2.76 (1.07)	3.90	3.90
*E. coli* WIBG 2.4	Ciprofloxacin	0.98	0.49	0.98
	Gentamicin	250	125	125
	Meropenem	0.98	0.98	0.69 (0.27)

*Bold type indicates a ≥ 4-fold change when comparing baseline sensitivities (P0) to P10 and X10 values. Data are expressed as geometric means from biologically duplicated experiments, with each comprising technical triplicates. Standard deviations are given in the parentheses if the data varied between replicates. Non-susceptible (ns) denotes no sensitivity breakpoint determined as the value was in excess of the antimicrobial concentrations used in the broth dilution.*

### Relative Pathogenicity of Passaged Bacteria

**FIGURE 1:**
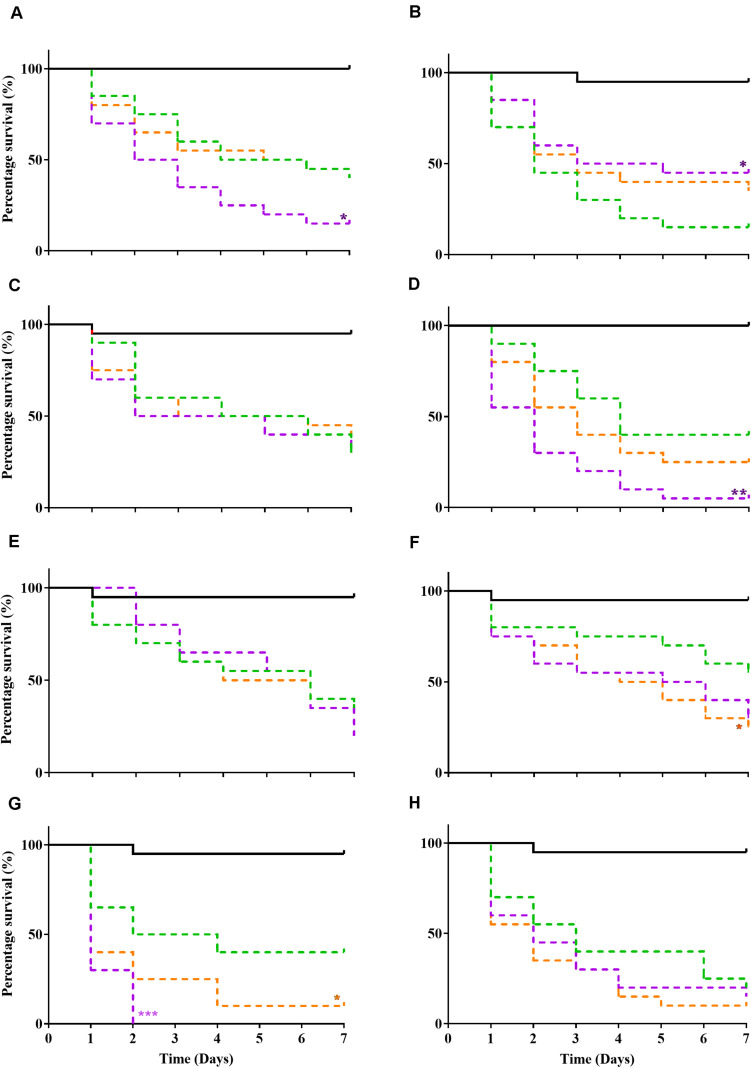
Kaplan Meir curves illustrating percentage survival following injection of *Galleria mellonella* (wax moth) with sterile PBS (solid black line), parent (P0, green dotted line), passaged (P10, purple dotted line), and X10 (orange dotted line) bacteria. Each curve represents a different test bacterium as follows: *S. aureus* WIBG 1.2 **(A)**, *S. aureus* WIBG 1.6 **(B)**, MRSA **(C)**, *S. epidermidis* **(D)**, *S. pyogenes* **(E)**, *E. coli* **(F)**, *P. aeruginosa* WIBG 1.3 **(G)**, *P. aeruginosa* WIBG 2.2 **(H)**. Significant differences in virulence following pairwise comparison with parent strain denoted as *, **, and *** (*P* ≤ 0.05, 0.01, and 0.001, respectively).

A *Galleria mellonella* waxworm model was used to determine relative pathogenicity in all tested bacteria (Figure 1). P10 passaged strains exhibited increased virulence (P ≤ 0.05, log-rank test) in 3/8 bacteria (*S. aureus* WIBG 1.2, *S. epidermidis* and *P. aeruginosa* WIBG 1.3; Figures 1A,D,G) when compared to parent strains (P0). These changes in pathogenicity were transient with partial or complete reversion in the absence of continued antimicrobial exposure (X10). A small but significant (*P* = 0.049) increase in larval killing was also observed in *E. coli* (X10) when compared to baseline data (Figure 1F). In contrast, a significant attenuation in pathogenicity was observed in the P10 of *S. aureus* WIBG 1.6 (Figure 1B).

### Impact of Manuka Honey Wound Gel Passaging on Bacterial Biofilm Formation

**FIGURE 2:**
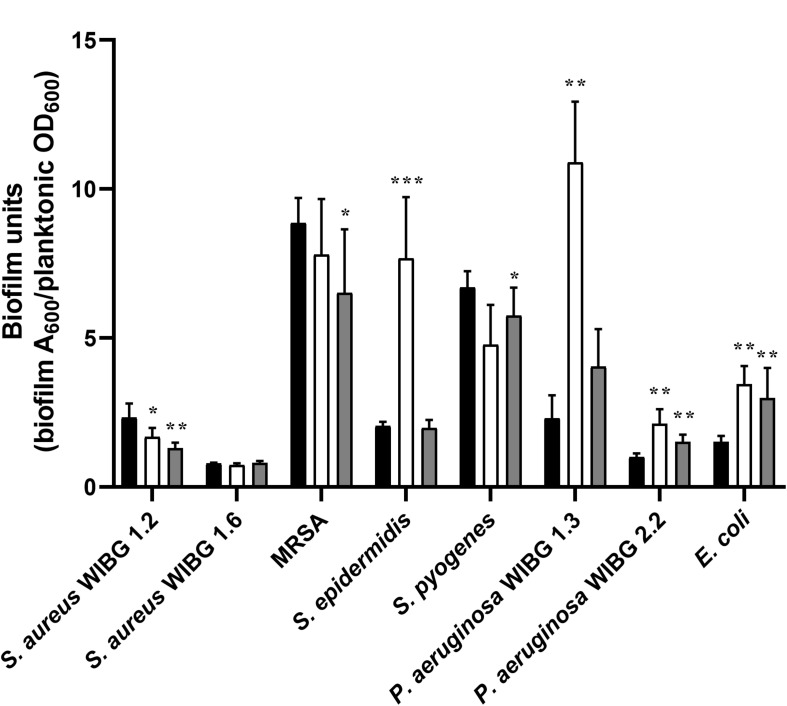
Biofilm formation in parent (P0, black) and passaged (P10, white; X10, dark grey) bacteria following adjustment for planktonic mass. Significant differences in biofilm formation following pairwise comparison with parent are denoted as *, **, and *** (*P* ≤ 0.05, 0.01, and 0.001, respectively). Error bars denote standard deviation.

A crystal violet assay was used to determine biofilm formation for all bacteria before and after repeated honey exposure and following ten passages in a honey-free medium (Figure 2). Overall, 3/8 strains exhibited significant reductions in biofilm formation following wound gel passaging (*S. aureus* WIBG 1.2, MRSA and *S. pyogenes*). In contrast, 4/8 strains, including *S. epidermidis*, *P. aeruginosa* (WIBG 1.3, 2.2) and *E. coli* showed a significant increase in biofilm formation according to the crystal violet assay. Reversion to baseline data was observed regarding biofilms formed by X10 *S. epidermidis* and X10 *P. aeruginosa* WIBG 1.3.

### Changes in Haemolytic Potential Following Passage

**FIGURE 3:**
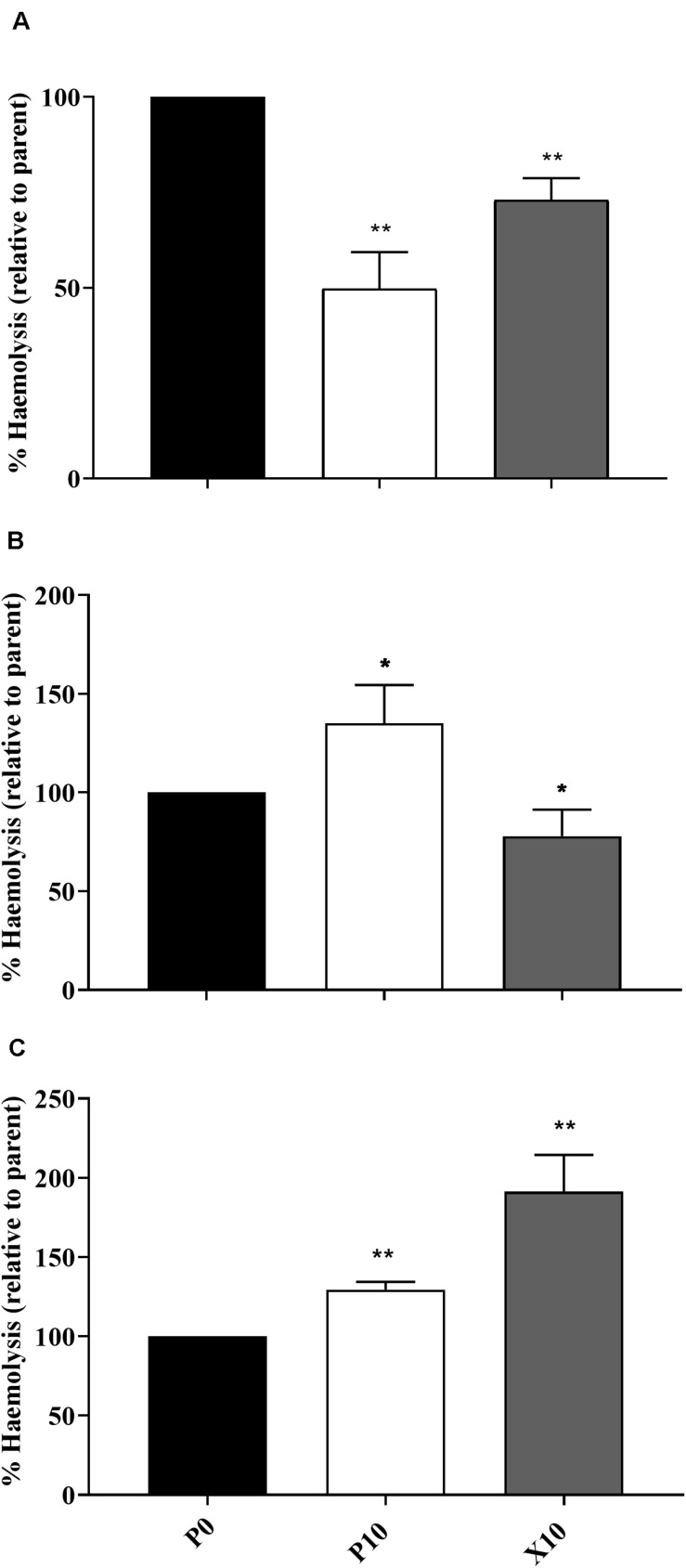
Haemolytic potential of parent bacterium (P0, black) and manuka honey wound gel passaged (P10, white; X10, dark grey) *S. aureus* WIBG 1.6 **(A)**, *S. epidermidis* **(B)** and *P. aeruginosa* WIBG 1.3 **(C)**. Data are relative to the haemolytic activity of the parent strain. Significant changes in haemolysis following pairwise comparison with baseline data are denoted as * and ** (*P* < 0.05 and 0.01, respectively). Error bars show standard deviation.

The ability of planktonic isolates to lyse erythrocytes was investigated in all passaged isolates that demonstrated: (i) a significant change in pathogenicity assay according to log-rank testing; (ii) observable haemolytic activity when incubated on blood supplemented agar and is illustrated in Figures 3A–C. In comparison to the parent, *P. aeruginosa* WIBG 1.3 (P10) showed a significant and sustained increase in haemolytic activity following passaging in the presence of manuka honey wound gel. Similar increases in haemolytic potential were also observed for *S. epidermidis*, although such observations were transient and marked by a small but significant reduction in haemolysis in strain X10. In contrast, attenuated haemolysis was noted in *S. aureus* WIBG 1.6 following wound gel passaging equivalent to 50% that of the progenitor strain.

### Modelling of Growth Curve Data

Of the eight strains examined for alterations in growth curve metrics, 5/8 showed significant changes in either carrying capacity, intrinsic growth rate or generation time after manuka honey wound gel exposure (Table 4). *S. aureus* WIBG 1.6, S. *aureus* WIBG 1.2 and *E. coli* exhibited a significant increase in both carrying capacity and doubling time with a significant decrease in intrinsic growth rate in both P10 and X10 bacteria. Additionally, *S. epidermidis* showed an increased doubling time concomitant to a reduction in intrinsic growth rate at X10. In contrast, *P. aeruginosa* WIBG 1.3 exhibited a significant and sustained decrease in both carrying capacity and doubling time following passaging.

**TABLE 4:** Growth curve metrics for parent and passaged bacteria.

Bacterium	Carrying capacity (k)			Growth rate (h^–1^)			Doubling time (h^–1^)		
	P0	P10	X10	P0	P10	X10	P0	P10	X10
*E. coli*	0.37 (0.07)	0.35 (0.03)	0.40 (0.04)	0.52 (0.07)	0.35 (0.02)**	0.37 (0.03)**	1.25 (0.16)	1.97 (0.10)**	1.78 (0.36)**
*P. aeruginosa* WIBG 1.3	0.52 (0.10)	0.30 (0.02)**	0.32 (0.03)**	0.54 (0.21)	1.44 (0.20)**	1.60 (0.36)**	1.43 (0.42)	0.49 (0.07)**	0.45 (0.10)**
*P. aeruginosa* WIBG2.2	0.44 (0.05)	0.36 (0.09)	0.41 (0.01)	0.77 (0.24)	1.18 (0.58)	0.83 (0.16)	1.06 (0.61)	0.79 (0.28)	0.87 (0.18)
*S. aureus* WIBG1.2	0.33 (0.08)	0.52 (0.04)**	0.55 (0.03)**	0.48 (0.05)	0.40 (0.02)**	0.40 (0.03)*	1.46 (0.19)	1.75 (0.11)**	1.73 (0.14)*
*S. aureus* WIBG1.6	0.49 (0.02)	0.60 (0.02)**	0.63 (0.06)**	0.43 (0.02)	0.37 (0.02)**	0.37 (0.03)*	1.60 (0.07)	1.89 (0.12)**	1.89 (0.17)*
MRSA	0.34 (0.03)	0.32 (0.07)	0.42 (0.05)	0.57 (0.15)	0.71 (0.09)	0.51 (0.26)	1.29 (0.31)	0.99 (0.13)	1.30 (0.29)
*S. epidermidis*	0.22 (0.02)	0.21 (0.01)	0.23 (0.03)	0.53 (0.04)	0.54 (0.06)	0.42 (0.04)**	1.31 (0.10)	1.30 (0.15)	1.66 (0.15)**
*S. pyogenes*	0.35 (0.02)	0.37 (0.01)	0.44 (0.14)	0.59 (0.13)	0.54 (0.11)	0.48 (0.14)	1.40 (0.42)	1.20 (0.01)	1.61 (1.06)

Significance denoted as *(P < 0.05) or **(P < 0.01) following pairwise comparison of P10 or X10 to baseline (P0) growth metric data. Standard deviations are given in the parentheses if the data varied between replicates.

### *In vitro* Coagulase Activity of Pasaged Staphylococci

The levels of coagulase produced by planktonic staphylococci were investigated using a tube coagulase test. After repeated passage with manuka honey wound gel, *S. aureus* WIBG 1.6 showed a delay in coagulation activity in strains P10 and X10, with both showing a positive result after 3 h compared to the parent, which exhibited a positive result after 30 min (Table 5). Both WIBG 1.2 and MRSA showed no observable change in coagulation over time. *S. epidermidis* (negative control) exhibited no observable coagulase activity.

**TABLE 5:** Coagulase activity of parent and passaged staphylococci.

Bacteria	Time (h)	Activity for passage		
		P0	P10	X10
*S. aureus* WIBG 1.2	0.5	++	++	++
	1	+++	+++	+++
	2	++++	++++	++++
	3	++++	++++	++++
*S.aureus* WIBG 1.6	0.5	++++	-	-
	1	++++	-	+
	2	++++	++	+++
	3	++++	+++ +	+ +++
MRSA	0.5	++	++	++
	1	+++	+++	+++
	2	++++	++++	++++
	3	++++	++++	++++
*S.epidermidis*	0.5	-	-	-
	1	-	-	-
	2	-	-	-
	3	-	-	-

*Tubes were observed for signs of coagulation over 3 h and scored on a five- point scale according to the manufacturer’s guidelines as follows: -, no coagulation detected; +, small separate clots; ++, small combined clots; +++, extensively coagulated clots; ++++, complete coagulation.*

### Pyocyanin Production in *P. aeruginosa*

**FIGURE 4:**
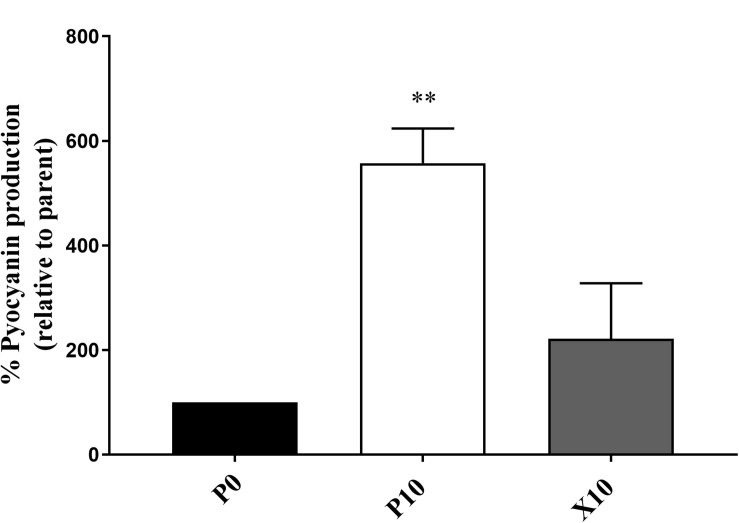
Pyocyanin production by *P. aeruginosa* WIBG 1.3 parent (P0, black) and manuka honey wound gel passaged (P10, white; X10, dark grey) bacteria. Data are expressed as a percentage respective to the progenitor (P0) strain. Significant data are represented as ** (*P* < 0.01). Error bar show standard deviation.

*P. aeruginosa* WIBG 1.3 showed a significant increase in the production of pyocyanin after repeated exposure to manuka honey wound gel (P0 vs. P10, 557.5% ± 66.3; *P* = 0.007). After removal of the antimicrobial challenge, a reversion in pyocyanin production was observed (P0 vs. X10, 221.5% ± 106.1; *P* = 0.19). Pyocyanin production was not observed in *P. aeruginosa* WIBG 2.2 (Figure 4).

## Discussion

The exposure of a test panel of bacteria to a wound gel resulted in both increases and decreases in antimicrobial susceptibilities. Overall, changes were relatively moderate (≤ 7.5% w/v) with regards to sensitivity to the manuka honey wound gel except for a sustained 1-fold increase in MIC in *S. epidermidis.* These observations are in keeping with previous research whereby stepwise resistance training in liquid culture was associated with only minor changes in sensitivity, although observed changes were transient (Cooper et al., 2010). The clinical significance of these observations is unclear with regards to their effect on therapeutic efficacy. The concentrations of manuka honey included in licensed wound care products are typically in excess of the sensitivities reported in the present study (Cooper et al., 2010). Wounds are, however, moist environments which may lead to variable product dilution, as has been discussed by Camplin and Maddocks (Camplin and Maddocks, 2014). Loss of activity following a pH mediated reduction in hydrogen peroxide production may also need to be considered (Bang et al., 2003; Molan and Rhodes, 2015; Cooper, 2016). The low MIC values reported from manuka honey sensitivity studies have been cited in support of the limited effects that wound dilution is likely to impart upon honey efficacy (Molan and Rhodes, 2015). Such effects are, however, less clear regarding the eradication of the biofilm phenotype from a wound environment.

Cross-resistance remains an area of concern regarding overuse of antimicrobials (Wales and Davies, 2015). Adaptation to suboptimal antimicrobial exposure may result in alterations in cell wall permeability or efflux systems that negatively impact upon antibiotic sensitivity profiles. To this end, the exposure of reference strains of *S. aureus* and *P. aeruginosa* to ciprofloxacin, tetracycline and oxacillin have been shown to rapidly generate antibiotic-resistant phenotypes but did not result in observable cross-resistance to honey (Blair et al., 2009). In the present study, whilst resistance to in-use concentrations of manuka honey were not observed, an additional aim was to investigate the effect of potential honey adaptation on antibiotic susceptibility. Changes in antibiotic profiles were limited, although it must be noted that an increase in MIC occured in *S. epidermidis* to erythromycin. Whilst these observations were transient (i.e.) the phenotype did not persist in the absence of further wound gel passaging, the increase was sufficient to cross a clinical breakpoint so that strain P10 was considered as resistant (The European Committee on Antimicrobial Susceptibility Testing, 2020). Trace levels of macrolides have been detected in honey previously (Bargańska et al., 2011), although, such findings are unlikely to be of relevance in formulations utilised in the healthcare setting given their controlled sourcing and rigorous processing procedures. As such, these data may suggest adaptive resistance via an unknown mechanism.

Changes in antimicrobial sensitivity following honey adaptation in biofilm growth modes have been reported previously (Camplin and Maddocks, 2014). Sessile communities of *P. aeruginosa* were exposed to Medihoney to determine changes in honey inhibitory concentrations, honey biofilm eradication concentrations and antibiotic sensitivities in the residing biofilm biomass. Overall, reduced sensitivities to both imipenem and rifampicin were observed in conjunction with marginal increases in planktonic and sessile honey sensitivities. Biofilms were not directly passaged in the present study but remain of interest as a future research direction. Rather, adapted planktonic cultures were investigated for subsequent changes in MBEC and biofilm-forming potential when tested using a crystal violet assay. Changes in biofilm eradication concentration were marginal in most cases, although a 4-fold reduction in gentamicin sensitivity was observed in a clinical isolate of *P. aeruginosa*. This observation occurred in conjunction with highly significant, but transient, increases in planktonic growth rate and biofilm formation, the later in support of previous investigations (Camplin and Maddocks, 2014).

The use of the *Galleria mellonella* waxworm model in this study enabled an assessment of the virulence potential of bacterial pathogens and suggested variable effects on pathogenesis, particularly between members of the staphylococci. In general, altered virulence in this genus occurred in conjunction with changes in growth metrics, haemolytic activity, coagulation and biofilm formation ability and agrees with studies investigating passaging in the presence of other antimicrobials (Latimer et al., 2012; Bazaid et al., 2018). Interestingly, the enhanced virulence observed in *P. aeruginosa* WIBG 1.3 (P10) is in contrast to previous reports. For example, *P. aeruginosa* wild-type PA14 has been shown to exhibit reduced pyocyanin production following exposure to both raw and heat-treated manuka honey, likely via interaction with the MvfR quorum sensing network (Wang et al., 2012). In the present study, honey adaptation was associated with significant increases in pyocyanin production following chloroform extraction. It must be noted that enhanced virulence may differ between strains as no significant changes in killing were observed in WIBG 2.2, supporting the view that honey is a complex compound comprising active elements capable of affecting multiple cellular target sites (Jenkins and Cooper, 2012). Whilst previous reports suggest the antimicrobial activity of honey to vary significantly between species, such observations may also be true for phenotypic adaptation between strains and warrants further investigation.

There are some limitations to this study that should be considered. Repeated growth in the absence of honey, analogous to the normal maintenance of bacteria in the laboratory, could conceivably alter bacterial physiology. The method we used has, however, been previously utilised successfully to address a range of research questions (Latimer et al., 2012; Forbes et al., 2014, 2019; Sun et al., 2018) and has been shown not to significantly alter antimicrobial susceptibility or biofilm formation where application without antimicrobial stress has been tested (Henly et al., 2019; Karmakar et al., 2019).

In summary, the repeated laboratory exposure of a test panel of bacteria to manuka honey, in the form of a wound gel, resulted in variable changes in both antimicrobial sensitivity and pathogenesis when compared to a progenitor. This is an important observation as chronic wounds provide an environment where antimicrobial wound dressings may be present *in situ* over prolonged periods of time. However, care must be taken in extrapolating the findings of an *in vitro* study to possible clinical effects. In the current study, phenotypic resistance to erythromycin was observed in a single wound isolate. Cross-resistance following manuka honey exposure has been rarely reported and ongoing work will elucidate the underlying mechanisms. It must be noted though that planktonic bacteria remained susceptible to concentrations of the manuka honey wound gel used in clinical applications, despite repeated exposures. Biofilm formation was also variably affected in passaged bacteria, with notable increases in the pseudomonads and *S. epidermidis.* Whilst this could have implications for treatment length, given the propensity for biofilms to form in wounds, significant changes in biofilm sensitivity were generally limited. With the exception of the pseudomonads and gentamicin, a favourable trend of marginal increases in biofilm antibiotic sensitivity were observed in most cases. The underlying mechanisms for such changes are not clear and warrant further investigation. This study also supports the role of additional phenotypic characterisation when investigating adaptation to antimicrobials.

## Data Availability Statement

The raw data supporting the conclusions of this article will be made available by the authors, without undue reservation.

## Author Contributions

GH and AM: conceptualisation. JM, GH, and VR: data curation and analysis. JM, GH, AM, VR, and RB: methodology. GH, AM, and RL: supervision. JM, GH, and AM: writing – original draft. GH, JM, AM, and RL: writing – review and editing. All authors contributed to the article and approved the submitted version.

## Conflict of Interest

GH held co-supervisory responsibilities for a Ph.D. studentship that is in-part funded by Matoke Holdings. AM conducts research and advises companies in the areas of antimicrobials, biofilms, microbiome, and microbial control. The remaining authors declare that the research was conducted in the absence of any commercial or financial relationships that could be construed as a potential conflict of interest.
